# Designing integrated early-phase protocols to reduce substantial modifications, including considerations for patient cohorts – a multi-stakeholder consensus view for a practical approach in Germany

**DOI:** 10.3389/fphar.2025.1699906

**Published:** 2026-01-26

**Authors:** R. Schultz-Heienbrok, S. Baumann, R. Böhm, A. Bonertz, K. Breithaupt-Groegler, U. Buckpesch-Heberer, M. Coenen, K. Erb-Zohar, N. Faisst, G. Grass, J. Höchel, A. Kovar, A. Muehlenbroich, J. Rengelshausen, C. Riedel, B. Schug, T. Sudhop, A. Warnke, B. Ziegele

**Affiliations:** 1 Charité Research Organisation GmbH, Berlin, Germany; 2 CRS Clinical Research Services Management, Berlin, Germany; 3 SocraTec R&D, Oberursel, Germany; 4 Universitätsklinikum Schleswig-Holstein, Kiel, Germany; 5 Paul-Ehrlich-Institut, Langen, Germany; 6 Consultant Clinical Pharmacology, Frankfurt, Germany; 7 Institute of Clinical Chemistry and Clinical Pharmacology, University Hospital Bonn, Bonn, Germany; 8 Clinphase, Schotten, Germany; 9 Dr. Falk Pharma GmbH, Freiburg, Germany; 10 Spezialisierte Ethikkommission für besondere Verfahren, Bonn, Germany; 11 Bayer AG, Berlin, Germany; 12 Sanofi, Frankfurt, Germany; 13 Novartis, Basel, Switzerland; 14 Uniklinik RWTH Aachen, Aachen, Germany; 15 Bundesinstitut für Arzneimittel und Medizinprodukte, Bonn, Germany

**Keywords:** clinical trials, complex trial design, first-in-human, integrated protocols, investigational medicinal product, substantial modification

## Abstract

• Frequent substantial modifications for initiating new trial parts slow down development and reduce transparency.

• Combining healthy volunteers and patients in a single early-phase trial raises safety, feasibility, and regulatory concerns.

• A multi-stakeholder consensus in Germany identified regulatory pathways to enable seamless transitions within integrated protocols for non-ATMP (advanced therapy medicinal product), non-oncology trials.

• The EMA FIH (first-in-human) guideline supports predefined transitions if scope, safety thresholds, and decision rules are clearly described.

• The German guardrail concept clarifies when transitions do not require a substantial modification, enhancing planning certainty.

• Actionable recommendations are provided on protocol design, decision criteria, and governance structures to enable early inclusion of patients without compromising safety or compliance.

• First positive experience has been gained with the presented framework in Germany.

## 1 Introduction

Combining separate smaller clinical trials into a single protocol has become common practice in early medicines development over the past decade (Erb-Zohar et al., 2018; Fruhner et al., 2017). For first-in-human trials (FIH), a single ascending dose (SAD) part followed by a multiple ascending dose (MAD) part is *de facto* the standard approach. This approach has benefits for sponsors and investigators:

- Sponsors benefit from unified planning and submission processes, and improved data integrity due to harmonised methodologies and centralised oversight. Adaptive designs allow for overlapping parts, streamlining development timelines. Cross-part pooling of placebo trial participants can further optimise sample sizes and statistical power.
- Investigators also benefit by becoming increasingly familiar with the investigational medicinal product (IMP) over the course of conducting several sub-trials, enabling more nuanced safety monitoring and interpretation of observations. Clinical trial participants in later phases benefit from this accumulated experience and oversight.

Despite these advantages, increasing complexity of early clinical trials poses significant challenges to ensure safety and wellbeing of trial participants and maintain data integrity and scientific validity. Furthermore, overly lengthy protocols impair readability and increase risks of implementation errors. Prolonged studies that continually add sub-parts via substantial modifications risk regulatory opacity, particularly regarding EU transparency requirements (European Medicines Agency, 2025). Timely publication of trial outcomes should be prioritised to enhance transparency and support informed decision-making among stakeholders, including patient advocacy groups. Thus, the wish for combining various sub-trials needs to be carefully balanced with ICH requirements for clarity and simplicity in design, conduct and presentation of a trial (ICH, 1995; ICH, 2021).

Also, sponsors struggle with ambiguity of when substantial modifications are required under EU Clinical Trial Regulation 536/2014 or expected by authorities when transitioning from one trial part to the next. Although the EMA FIH guideline (European Medicines Agency, 2017) provides core principles for addressing such questions, it leaves room for interpretation—especially regarding patient inclusion, governance structures, and transition criteria within integrated protocols.

In response, German regulatory authorities have proposed—based on the experience with SAD/MAD transitions in healthy volunteers—the “guardrail concept” (BfArM and PEI, 2024) to define protocol-internal boundaries that allow progression without requiring new approvals. This paper seeks to expand on the guidance of this guardrail concept by consolidating regulatory and ethical expectations into a coherent framework with three key components: (1) defining the permissible scope of integrated protocols, (2) establishing robust, data-driven criteria for transitions between trial parts, and (3) specifying governance processes for transitioning to new trial parts.

By providing actionable, consensus-based recommendations, this brief supports sponsors in designing compliant, flexible, and patient-inclusive early-phase trials. The framework presented here reflects current best practices and the regulatory consensus in Germany derived from a meeting in Bonn in December 2023 (AGAH Discussion Forum with participants from Regulatory Authorities, Ethics Committees, Research Organisations and Sponsors) around the two aspects of handling substantial modifications and the possibility of including patients in such trials.

## 2 Policy review on combined protocols

The key guideline for integrating sub-studies into a single protocol in Europe is the “Guideline on strategies to identify and mitigate risks for first-in-human (FIH) and early clinical trials with investigational medicinal products” (European Medicines Agency, 2017), further on referred to as “EMA FIH” in this text. The 2017 revision introduced integrated protocols, primarily to guide transitions from SAD to MAD in healthy volunteers. The scope of EMA FIH only covers clinical trials with new chemical and biotechnological entities. ATMP (advanced therapy medicinal product) and oncology trials are outside this paper’s scope.

EMA FIH outlines three critical elements which must be defined in an integrated early-phase trial design: (1) the scope of what can be combined in a single protocol, (2) the criteria for progressing from one trial part to the next, and (3) the decision-making process and responsible parties. These steps form the regulatory and operational backbone for constructing complex protocols that include different populations or treatment regimens while ensuring participant safety and regulatory compliance.

### 2.1 Scope: What can be combined in a single protocol?

EMA FIH does not restrict which or how many trial parts might be combined. The guideline generally covers trials on safety, tolerability, PK (pharmacokinetics) and PD (pharmacodynamics). Section 2 of the guideline explicitly mentions that the following research interests might be included in such a trial: “These trials may also include collection of data on, e.g., food or drug interactions, different age groups or gender, proof of concept and relative bioavailability of different formulations”. The guideline acknowledges that such trials may involve both healthy volunteers and patients. It states: “These trials are often undertaken in healthy volunteers but can also include patients.” Thus, combining populations is acceptable if justified. EMA FIH requires that “all parts (…) should be predefined within an integrated protocol” (Section 8.2.2), including the maximum number of participants, dosing regimens, and transition criteria.

Additional limitations are drawn from the Clinical Trials Coordination Group (CTCG), which calls for an overarching scientific hypothesis to tie all parts together (CTCG, 2019). ICH E8 (ICH, 2021) adds that protocols must remain operationally feasible and should avoid unnecessary complexity, reinforcing the need to limit scope to what is feasible and meaningful.

Thus, a protocol combining multiple dose regimens, populations, or sub-studies is acceptable if:

- the scope is well-justified by a unifying hypothesis,
- the design avoids avoidable complexity, and
- each component’s contribution to the overarching research question is clearly described.

This understanding of scope aligns with ICH E8(R1), Section 4.3.1, which defines Human Pharmacology Studies as including PK, PD, safety, tolerability and—where appropriate—exploratory efficacy. Consolidating these elements within a single, methodologically coherent protocol is therefore both scientifically and regulatorily justified.

### 2.2 Decision criteria for progressing between trial parts

Transitions between trial parts—such as from SAD to MAD or from healthy volunteers to patients—must be based on predefined, data-driven decision rules. In line with EMA FIH Sections 7.4, 8.2.2 and 8.2.9, the protocol should define dose escalation criteria, stopping rules and criteria guiding the decision to progress from one trial part to another.

These rules must be based on the totality of data, including:

- Safety data (e.g, adverse events (AEs), clinical laboratory parameters, vital signs, ECG)
- Pharmacokinetic (PK) data to assess exposure and compare it to nonclinical predictions
- Pharmacodynamic (PD) data, if relevant and available

Section 7.3 of the guideline further requires that for patient populations, the starting dose is expected to have a minimal pharmacological effect and must be safe to use. In contrast, for healthy participants, the starting dose should stay below predicted PAD (pharmacologically active dose) exposure.

The guideline emphasises that escalation steps should be conservative and justified, especially once a pharmacologically active range has been reached (EMA FIH, Sections 7.4, 7.5). Based on the consensus process reported in this article, when pharmacodynamic activity has been reached, dose increments above a two-fold increase should be avoided unless appropriately justified.

The protocol should outline specific “stopping rules” that require dosing or progression to pause. Examples include:

- Occurrence of serious or severe AEs judged related to the IMP
- Safety signals in laboratory, ECG, or clinical parameters crossing predefined thresholds
- Exposure levels exceeding pre-established PK limits

These rules act as guardrails for risk management. Clear documentation of such rules in the protocol facilitates timely decisions and regulatory alignment, especially in complex early clinical trials where flexibility must not compromise safety.

Optional parts and flexible design elements (e.g., optional cohorts) must also follow predefined boundaries. In line with EMA FIH sections 8.2.2 and 8.2.8, all parts and options within an integrated protocol should be predefined, and the data to be used to make decisions about proceeding should be clearly described.

### 2.3 Governance of trial part transitions

EMA FIH underlines the need for a defined decision-making process. Typically, a decision-making group (DMG) evaluates whether criteria are met for transitions. Its composition (e.g., principal investigator (PI), sponsor representatives, possibly external experts) should be stated in the protocol.

EMA FIH does not mandate the use of an independent Data Monitoring Committee (DMC) (European Medicines Agency, 2005) for early-phase trials. The German authorities BfArM (Bundesinstitut für Arzneimittel und Medizinprodukte/PEI (Paul-Ehrlich Institute) have addressed this in their national recommendations for complex protocols (BfArM and PEI, 2024): it supports context-sensitive governance structures adapted to trial complexity and participant vulnerability.

In practice, decisions must follow:

- Defined criteria,
- Documented procedures, and
- A DMG

While EMA FIH provides well-defined criteria for transitions between SAD and MAD parts in healthy volunteers, its guidance becomes less specific when considering the integration of patient cohorts into early-phase protocols. Nevertheless, the underlying principles—namely, the requirement for predefined boundaries, data-driven progression rules and clear governance—remain applicable.

Inclusion of patients is not a default option but requires careful justification by the absence of suitable PD readouts in healthy trial participants, the availability of an acceptable safety margin or the prospect of direct clinical benefit. When these conditions are met, the same framework that governs escalation and transition decisions in healthy volunteers can be extended to patient populations.

Similarly, the concept of predefined protocol limits—guardrails—applies also to broader transitions between trial parts. Section 8.2.2 of the EMA FIH guideline establishes that substantial modifications are required when changes exceed these predefined boundaries. This includes, for example, exceeding maximum exposure limits, introducing major alterations in escalation strategy or adjusting eligibility or stopping rules.

Accordingly, sponsors must define boundaries clearly to distinguish in-protocol adaptations from those requiring prior approval. The next section “Actionable recommendations” outlines how to operationalise these principles—ensuring scientific, ethical and regulatory robustness.

While EMA FIH is the most fundamental document for designing early-phase clinical trials, other guidance documents must also be considered for compliance and adequacy. These include but are not limited to:

- Substance-specific guidelines (e.g., for monoclonal antibodies or small molecules) addressing pharmacology and risks.
- Indication-specific guidelines on clinical efficacy and safety requirements, published by the EMA (Clinical efficacy and safety guidelines, European Medicines Agency).

These additional guidelines can impose specific requirements for patient selection, monitoring, and risk mitigation and must align with protocol design. Other general guidelines, such as ICH E8 (ICH, 2021), ICH E20 (ICH, 2025) and CTCG recommendations (CTCG, 2019) also offer supportive design guidance.

**FIGURE 1:**
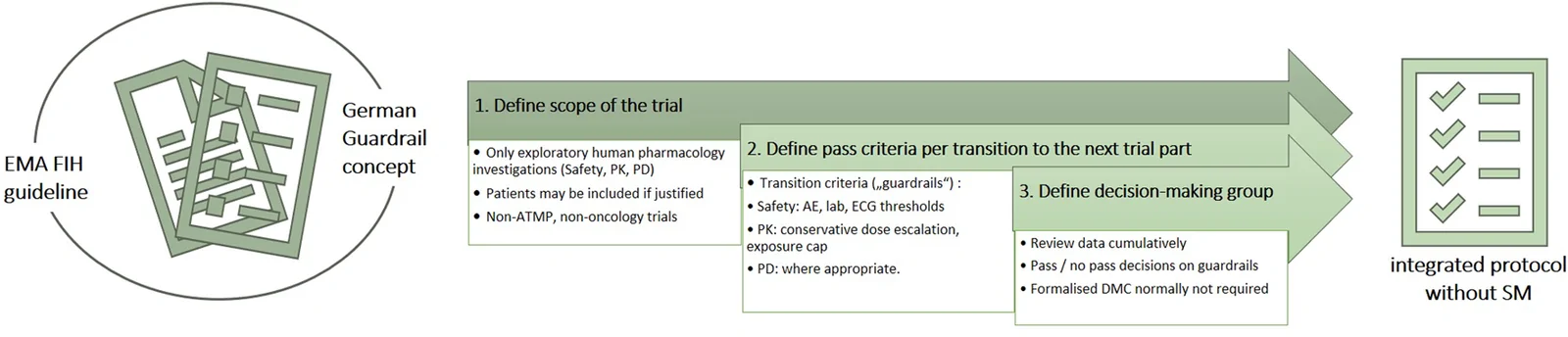
Flowchart illustrating the German Guardrail concept within the context of EMA FIH guideline. It includes three main steps: defining trial scope, establishing transition pass criteria, and forming a decision-making group. The process leads to an integrated protocol without substantial modification.

The three elements of an integrated protocol—definition of scope, decision criteria, and decision-making group—are summarised in Figure 1. While this framework is well established for transitions between SAD and MAD in healthy volunteers, its applicability to patient populations without requiring a substantial modification depends on the strength of justification and the robustness of predefined decision criteria. The following section outlines the key elements for developing such justifications and for formulating appropriate decision criteria.

## 3 Actionable recommendations

The multi-stakeholder discussion focused on practical strategies for designing and conducting integrated early-phase clinical trials, particularly those that include patient populations. The German regulatory authorities BfArM and PEI have recently published guidance on transitioning to patient-involving trial parts, which further inform this section (BfArM and PEI, 2024). The recommendations below provide concrete advice on how to present trial part transitions and justify decision-making processes within an integrated clinical trial protocol. It is acknowledged that each trial must ultimately be assessed on a case-by-case basis, applying the ‘totality of data’ principle outlined in the EMA FIH guideline. In this ‘totality of data’ context, non-clinical data or IMP characteristics might be understood as “overarching guardrails”. The recommendations in this section are therefore not prescriptive requirements, but serve as a practical toolkit to support justification and promote consistency in integrated early-phase designs.

### 3.1 Trial design and decision process in the protocol

Commonly used protocol templates (e.g., TransCelerate (Gill, 2014)) lack dedicated sections summarising the design logic and decision processes governing trial part progression. However, ICH M11 (currently in Step 2b (ICH, 2022)) recommends consolidating this information in Section 4.1, “Description of the Trial Design,” with further justifications to be presented in Section 4.2.

To improve clarity and transparency, we recommend summarising key decision points and criteria using structured formats such as bullet points or tables. Table 1 outlines the typical decision points that should be addressed, along with requirements for minimum datasets, evaluation timelines, and responsible parties.

**TABLE 1:** Considerations for possible decision points in an integrated early phase trial.

1. Key decision points	
Decision point	Explanation
Sentinels	Sentinel dosing in any cohort is generally considered appropriate and implies dosing of the active IMP (investigational medicinal product) in one participant, followed by an observation period before dosing the next. Deviations must be risk-justified in the protocol
Dose escalation	In line with EMA FIH’s (first-in-human), focus on exposure rather than absolute doses. Dose steps and maximum dose must be clearly linked to objectives (e.g., systemic exposure) and justified. Increments >2-fold above biologically active dose need thorough justification
Transition to new trial part	Requires pre-defined and unambiguous conditions to proceed without a substantial modification
Optional cohorts or trial parts	Planned options and conditions for adding or dropping parts must be specified
Cohort expansion	Optional expansions must be pre-specified and justified
Addition or shift of data collection timepoints	If emerging data (e.g., PK/biomarkers) suggest suboptimal timing, the protocol must define under which conditions adjustments are allowed

2. Minimum dataset per decision point	
Data	Details
Safety data	Always required. Includes AE grading, physical examination, vital signs, clinical laboratory and ECG. Additional assessments based on, e.g., population, safety profile, MoA (mechanism of action) to be considered. Multi-centre trials must define inter-site communication
PK data	Expected unless justified. Required to link safety findings to exposure
PD data	Should be considered. Plateauing PD response may limit further escalation
Time after last dosing	Cut-off for data collection must be stated. Sentinel focus t_max_, escalation/part transition PK/PD-driven
Evaluable trial participants	Cumulative assessment expected. If incomplete data are used for decision, this must be justified in the protocol

3. Additional considerations	
Aspect	Details
Tailoring of criteria	Criteria should reflect MoA, non-clinical evidence, population, and trial phase. EMA FIH minimum standards apply
Cut-off values and lab re-tests	Must be pre-defined, including rules for re-measurement and context-sensitive interpretation
Multiple populations	Separate criteria may apply per population; to be reflected in stopping rule section
Decision maker	Must be named for each decision point: PI (principal investigator), PI + sponsor, or defined decision-making group (e.g., for escalation/transition). No decision may override the PI

Staggering refers to spacing of dosing within a given cohort after sentinel dosing. There are two reasons for staggering:

- Safety staggering should be considered as an additional risk mitigation tool, particularly in substances with a pharmacological risk profile that suggests the potential for early-onset serious adverse events (SAEs) or clinically relevant warning signals. Dosing is spaced to allow sufficient observation time before proceeding with subsequent participants. Staggering in this sense functions as an additional decision point in the trial. The pharmacological rationale, safety signals to be monitored, and the duration of observation would then be outlined in the protocol.
- Organisational staggering refers to the spacing of dosings to ensure that sites can adequately respond to AEs occurring in close temporal proximity. This approach is driven by logistical capacity and ensures that staff and infrastructure would not be overburdened if intervention-requiring events occurred. This type of staggering is typically not outlined in the protocol but part of general suitability assessment of the trial site.

In FIH trials, safety and tolerability constitute the primary endpoints, and safety criteria underpin all key decisions. Accordingly, the grading of AE severity must be carefully justified. Grading systems should consider the required level of intervention, symptom tolerability, and impact on daily activities, and must extend beyond symptomatic AEs to include laboratory abnormalities and vital signs. Definitions should provide operational clarity while allowing sufficient flexibility to account for clinical context—for example, the same laboratory deviation may warrant different actions depending on the trial population’s vulnerability. Protocols should specify when confirmation of out-of-range laboratory results is required with analysis of a new sample. While sponsors may define study-specific grading criteria, established references such as the FDA toxicity grading scale for healthy volunteers (FDA, 2024) and the CTCAE (National Cancer Institute, 2017) may offer suitable frameworks.

### 3.2 Combining different populations

Before initiating a new trial part—particularly when transitioning from healthy volunteers to patient cohorts—it is essential that the preceding part has been sufficiently evaluated. Sponsors should ensure that all relevant safety, PK, and, where applicable, PD data have been collected, reviewed, and interpreted in line with the predefined criteria before proceeding to the next part. This sequential evaluation safeguards participants, upholds data integrity, and aligns with both regulatory expectations and ethical standards.

Including patient populations alongside healthy volunteers is increasingly explored in early-phase trials. When justified by the limitations of PD data in healthy trial participants, such integration can add valuable insights—but requires clear risk-benefit reasoning, appropriate safeguards, and a well-defined therapeutic context. Especially the specific patient risk arising from a lack of efficacy should be duly addressed when justifying the inclusion of a patient population.

Table 2 outlines key considerations and criteria for justifying inclusion of a patient population in the trial to support clinical trial planning.

**TABLE 2:** Considerations for target populations in early clinical trials.

Key points for justifying the scientific and clinical rationale
PD response in healthy trial participants not adequately assessable
Working within therapeutic dose range
Disease progress/stability of baseline data
Consequences of lack of efficacy
Possible differences in PK and safety profile between populations
Consideration of disease grade
Uncertainties with the IMP (investigational medicinal product) (first in class, limited safety data)
Ability to assign AEs to disease or IMP
Risk mitigation and study design considerations
Suspected adverse drug reaction (ADR) profile
Risks deriving from the IMP with regard to PK predictability and activity/duration of action
Burden from trial procedures (often more demanding in early safety trials)
Need for pausing baseline therapy (‘*do not harm*’)
Co-medication use in patient population
Protocol and operational implications
Recruitability of trial participants
Meaningful definition of population through in-/exclusion criteria
Consideration of disease grade
Ethical and regulatory aspects
Qualification of the investigator, expertise with patient condition at the site
Informed consent adapted to specific patient risks
Predefinition of safety criteria and their application in patients
Clearly defined and documented responsibilities at site level

### 3.3 Route of administration: i.v. versus s.c.

The use of subcutaneous (s.c.) instead of intravenous (i.v.) route of administration is sometimes regarded as additional risk and therefore was discussed as well during the consensus meeting in the context of cohort/part transitioning requirements. Usually, in case of biologics, starting with an i. v. infusion is regarded as safer. Starting directly with s. c. administration, however, can be justified under certain conditions. While i. v. offers predictability and real-time control, s. c. may align better with development goals. Regulatory consensus now acknowledges that a mandatory i. v. lead-in is not always necessary when the considerations in Table 3 are being adhered to.

**TABLE 3:** Considerations for i.v./s.c administration in early clinical trials with biologics.

Topic	Consideration
i.v. requirement	For new medicines intended for s.c. administration only, there is no mandatory requirement for investigating the i.v. administration before the s.c. route is explored
Justification of s.c. only	It should be clearly described and justified in the protocol if and why the sponsor considers s.c. arms sufficient to address all relevant research questions
Need for i.v. arm(s)	The potential need of i.v. arm(s) should be derived from the specific questions to be addressed in the trial
PK relevance	For many clinically relevant PK and PK/PD-related questions, s.c. data—also in combination with nonclinical data and modeling—can replace i.v. clinical data
Dose selection	To account for unknown bioavailability after s.c. administration, doses should be selected assuming 100% bioavailability, unless otherwise justified
Dose escalation	Dose selection and escalation must consider the expected variability of exposure to mitigate risks at each step. Greater variability with s.c. does not require larger cohorts, as sizes are not based on formal sample size calculation
Safety monitoring	Planned safety monitoring and decision points must reflect PK/PD specificities of the chosen route (e.g., later t_max_ with s.c.).
Modeling and simulation	If meaningful and available, modeling and simulation summaries should be provided to support justification and facilitate protocol review

### 3.4 Involvement of independent experts

Integrated early-phase trials may not formally require an independent data monitoring committee (IDMC/DSMB), particularly in Germany. However, in scenarios involving vulnerable populations, long trial durations, or partially unblinding outcomes, the involvement of external expert reviewers can enhance transparency, safety oversight, and public trust.

Table 4 outlines specific conditions under which independent expert involvement should be considered. These are not regulatory requirements but reflect best practices drawn from both regulatory interpretation and stakeholder consensus.

**TABLE 4:** Conditions when involving independent experts should be considered.

Condition	Rationale
Trial duration and frequency of multiple dosing	Longer or repeated exposure increases risk; expert oversight supports ongoing risk assessment; independent interim analyses
Inclusion of patients (the more vulnerable, the higher the need for additional safety monitoring)	Vulnerable populations may require enhanced protection through independent expert input
In case observed effects unblind the trial team (e.g., certain adverse effects or PD reactions)	Independent experts can help interpret unblinded data objectively and manage potential bias
Assessments which need specialists to evaluate	Complex or specialised assessments benefit from external expertise to ensure accuracy and safety
Criticality of mechanism of action/treatment	High-risk or novel mechanisms may require external review to evaluate unexpected findings
Critical events (pause of the trial) or critical trial part transitions	Independent experts provide objective judgement during trial interruptions or key progression steps

### 3.5 Substantial modifications

A recurring concern for sponsors is whether the initiation of a new trial part constitutes a substantial modification. The German guardrail approach addresses this by allowing predefined boundaries for progression without triggering new submissions. The guardrails must be clearly described in the protocol and respected throughout.

Table 5 provides practical recommendations on how to define such boundaries for both regulatory and ethical oversight, including guidance on updating participant information and determining when a formal modification is required.

**TABLE 5:** Practical considerations: substantial modifications.

Topic	Details
Protocol-defined boundaries	For trial part transitions, the protocol should clearly define the boundaries beyond which a substantial modification is required
Typical cases triggering substantial modifications (European Medicines Agency, 2017; European Commission, 2014; European Commission, 2025)	– Observed exposure exceeds approved maximum exposure – Steeper dose escalations than stipulated in the trial protocol – Events impacting the reference safety information – New data changing the risk-benefit assessment – Changes to the sample size – Changes to inclusion or exclusion criteria – Changes to discontinuation criteria/stopping rules – Changes in dose levels outside predefined decision criteri
Divergence of guardrails	Guardrails might differ between the national competent authority and the ethics committee
Participant information updates without re-assessment	The protocol should state the boundaries within which participant information can be updated for a new trial part without re-assessment
Typical updates covered by ‘EC guardrails’	– Type, number and severity of related AEs – Doses, including highest dose, administered to humans – Number of participants treated with the IMP (investigational medicinal product) – Description of overall tolerability of the IMP
Principle of dynamic integration	Participant information must keep pace with the frequent integration of new information in a complex trial
Protocol-based information update rules	Integration of new information into the participant information must follow clear rules, outlined in the protocol

## 4 Conclusion

Integrated early-phase clinical trials that combine healthy participants and potential patient cohorts under a single protocol represent a modern and pragmatic approach to drug development. When properly planned and guided by predefined decision criteria, such designs can generate pharmacodynamic insights earlier in the development timeline, accelerate progression to later phases, and reduce the administrative burden caused by frequent substantial modifications.

EMA FIH provides a robust regulatory framework for these trials. However, as this paper highlights, additional clarification is needed—particularly regarding the integration of patient populations and the conditions under which transitions between trial parts do not require substantial modifications. The recently articulated German “guardrail” approach offers valuable guidance in this regard and may serve as a model for broader implementation.

By incorporating these principles and applying the actionable recommendations outlined in this policy brief, sponsors can uphold trial integrity and participant safety while gaining efficiencies in design and execution. Embracing this consensus-driven approach will greatly facilitate approval and execution of early clinical trials in Germany, and has already been accepted by authorities in selected cases involving patient cohorts without the need for substantial modifications.
