# Correction: Designing integrated early-phase protocols to reduce substantial modifications, including considerations for patient cohorts – a multi-stakeholder consensus view for a practical approach in Germany

**DOI:** 10.3389/fphar.2026.1948963

**Published:** 2026-09-02

**Authors:** R. Schultz-Heienbrok, S. Baumann, R. Böhm, A. Bonertz, K. Breithaupt-Groegler, U. Buckpesch-Heberer, M. Coenen, K. Erb-Zohar, N. Faisst, G. Grass, J. Höchel, A. Kovar, A. Muehlenbroich, J. Rengelshausen, C. Riedel, B. Schug, T. Sudhop, A. Warnke, B. Ziegele

**Affiliations:** 1 Charité Research Organisation GmbH, Berlin, Germany; 2 CRS Clinical Research Services Management, Berlin, Germany; 3 SocraTec R&D, Oberursel, Germany; 4 Universitätsklinikum Schleswig-Holstein, Kiel, Germany; 5 Paul-Ehrlich-Institut, Langen, Germany; 6 Consultant Clinical Pharmacology, Frankfurt, Germany; 7 Institute of Clinical Chemistry and Clinical Pharmacology, University Hospital Bonn, Bonn, Germany; 8 Clinphase, Schotten, Germany; 9 Dr. Falk Pharma GmbH, Freiburg, Germany; 10 Spezialisierte Ethikkommission für besondere Verfahren, Bonn, Germany; 11 Bayer AG, Berlin, Germany; 12 Sanofi, Frankfurt, Germany; 13 Novartis, Basel, Switzerland; 14 Uniklinik RWTH Aachen, Aachen, Germany; 15 Bundesinstitut für Arzneimittel und Medizinprodukte, Bonn, Germany

**Keywords:** clinical trials, complex trial design, first-in-human, integrated protocols, investigational medicinal product, substantial modification

In the published article there were three instances of incorrectly used quotation marks although the wording paraphrased the guideline rather than quoting it verbatim. In addition, one incorrect section reference to the EMA FIH guideline has been identified.

A correction has been made to section 2.2, paragraph 1. In the published article, the text reads:

According to EMA FIH (Section 8.2.1): “The protocol should define dose escalation and stopping rules and other criteria guiding the decision to progress from one part of the trial to another.”

This has been corrected to read:

In line with EMA FIH Sections 7.4, 8.2.2 and 8.2.9, the protocol should define dose escalation criteria, stopping rules and criteria guiding the decision to progress from one trial part to another.

A correction has been made to section 2.2, paragraph 2. In the published article, the text reads:

Section 6.7 of the guideline further requires that for patient populations, the starting dose is expected to have a minimal pharmacological effect and must be safe to use

This has been corrected to read:

Section 7.3 of the guideline further requires that for patient populations, the starting dose is expected to have a minimal pharmacological effect and must be safe to use.

A correction has been made to section 2.2, paragraph 3. In the published article, the text reads:

The guideline emphasises that escalation steps should be conservative and justified, especially once a pharmacologically active range has been reached. It explicitly advises that “when pharmacodynamic activity has been reached, dose increments above a two-fold increase should be avoided unless appropriately justified” (EMA FIH, Section 8.2.2).

This has been corrected to read:

The guideline emphasises that escalation steps should be conservative and justified, especially once a pharmacologically active range has been reached (EMA FIH, Sections 7.4, 7.5). Based on the consensus process reported in this article, when pharmacodynamic activity has been reached, dose increments above a two-fold increase should be avoided unless appropriately justified.

A correction has been made to section 2.2, paragraph 5. In the published article, the text reads:

Optional parts and flexible design elements (e.g., optional cohorts) must also follow predefined boundaries. As stated in Section 8.2.2: “All parts and options within an integrated protocol should be predefined, and the data to be used to make decisions about proceeding should be clearly described.”

This has been corrected to read:

Optional parts and flexible design elements (e.g., optional cohorts) must also follow predefined boundaries. In line with EMA FIH sections 8.2.2 and 8.2.8, all parts and options within an integrated protocol should be predefined, and the data to be used to make decisions about proceeding should be clearly described.

The original version of this article has been updated.

